# Epigenome-wide association study of lung function in Latino children and youth with asthma

**DOI:** 10.1186/s13148-022-01227-5

**Published:** 2022-01-15

**Authors:** Esther Herrera-Luis, Annie Li, Angel C. Y. Mak, Javier Perez-Garcia, Jennifer R. Elhawary, Sam S. Oh, Donglei Hu, Celeste Eng, Kevin L. Keys, Scott Huntsman, Kenneth B. Beckman, Luisa N. Borrell, Jose Rodriguez-Santana, Esteban G. Burchard, Maria Pino-Yanes

**Affiliations:** 1 Genomics and Health Group, Department of Biochemistry, Microbiology, Cell Biology and Genetics, Universidad de La Laguna, Apartado 456, 38200 San Cristóbal de La Laguna, Santa Cruz de Tenerife Spain; 2 Department of Medicine, University of California San Francisco, San Francisco, CA USA; 3 Berkeley Institute for Data Science, University of California Berkeley, Berkeley, CA USA; 4 UMN Genomics Center, Minneapolis, MN USA; 5 Department of Epidemiology and Biostatistics, Graduate School of Public Health and Health Policy, City University of New York, New York, NY USA; 6 Centro de Neumología Pediátrica, San Juan, PR USA; 7 Department of Bioengineering and Therapeutic Sciences, University of California San Francisco, San Francisco, CA USA; 8 CIBER de Enfermedades Respiratorias, Instituto de Salud Carlos III, Madrid, Spain; 9 Instituto de Tecnologías Biomédicas (ITB), Universidad de La Laguna, San Cristóbal de La Laguna, Tenerife Spain

**Keywords:** Lung function, Latinos, Hispanics, Asthma, Epigenome-wide association study, Methylation

## Abstract

**Introduction:**

DNA methylation studies have associated methylation levels at different CpG sites or genomic regions with lung function. Moreover, genetic ancestry has been associated with lung function in Latinos. However, no epigenome-wide association study (EWAS) of lung function has been performed in this population. Here, we aimed to identify DNA methylation patterns associated with lung function in pediatric asthma among Latinos.

**Results:**

We conducted an EWAS in whole blood from 250 Puerto Rican and 148 Mexican American children and young adults with asthma. A total of five CpGs exceeded the genome-wide significance threshold of *p* = 1.17 × 10^−7^ in the combined analyses from Puerto Ricans and Mexican Americans: cg06035600 (*MAP3K6*, *p* = 6.13 × 10^−8^) showed significant association with pre-bronchodilator Tiffeneau–Pinelli index, the probes cg00914963 (*TBC1D16*, *p* = 1.04 × 10^−7^), cg16405908 (*MRGPRE*, *p* = 2.05 × 10^−8^)*,* and cg07428101 (*MUC2*, *p* = 5.02 × 10^−9^) were associated with post-bronchodilator forced vital capacity (FVC), and cg20515679 (*KCNJ6*) with post-bronchodilator Tiffeneau–Pinelli index (*p* = 1.13 × 10^−8^). However, these markers did not show significant associations in publicly available data from Europeans (*p* > 0.05). A methylation quantitative trait loci analysis revealed that methylation levels at these CpG sites were regulated by genetic variation in Latinos and the Biobank-based Integrative Omics Studies (BIOS) consortium. Additionally, two differentially methylated regions in *REXOC* and *AURKC* were associated with pre-bronchodilator Tiffeneau–Pinelli index (adjusted *p* < 0.05) in Puerto Ricans and Mexican Americans. Moreover, we replicated some of the previous differentially methylated signals associated with lung function in non-Latino populations.

**Conclusions:**

We replicated previous associations of epigenetic markers with lung function in whole blood and identified novel population-specific associations shared among Latino subgroups.

**Supplementary Information:**

The online version contains supplementary material available at 10.1186/s13148-022-01227-5.

## Introduction

Asthma is the most prevalent chronic inflammatory disorder among children and young adults worldwide [1]. It is characterized by reversible airflow obstruction and impaired lung function, with a larger decline in lung function among children and adults with asthma than in individuals without asthma [2]. Although many genotypic predictors of respiratory diseases have been identified, genetic effects do not fully explain the high prevalence of asthma worldwide [3]. Decreased lung function in childhood and early adulthood is an important predictor of wheezing [4], asthma severity [5], future reduced lung function [6], the development of chronic obstructive pulmonary disease (COPD), and even death [7]. Furthermore, lung function is also influenced by several factors, including smoking exposure, air pollution, socioeconomic factors, and prenatal exposures [8, 9]. These environmental factors can observably impact the epigenome.

The most studied epigenetic marker type is DNA methylation (DNAm), which consists of the addition of a methyl group to cytosine residues within 5′-cytosine-phosphate-guanine-3′ dinucleotide sequences (known as ‘CpG’ sites). Changes in DNAm can regulate gene function by modulating gene expression in response to a wide range of environmental factors or genetic determinants [10]. The association between different methylation patterns and lung function can be analyzed with epigenome-wide association studies (EWAS). However, all EWAS of lung function have been conducted in European-descent populations, except for a study in Korean adults with COPD [11].

The Hispanic/Latino populations are the largest minority group in the United States (US) [12]. Hispanics/Latinos are genetically diverse, with varying proportions of European, African, and Native American ancestries depending on each subgroup-specific historical event of genetic admixture [13]. Native American ancestry is associated with higher lung function in Mexican American children with asthma [14, 15], whereas African ancestry is associated with lower lung function among Mexican American and Puerto Rican children with asthma [15, 16].

Since methylation is also associated with genetic ancestry [17], we hypothesize that differential DNAm patterns in whole blood may contribute to the differences in lung function among Puerto Ricans and Mexicans. Therefore, we aimed to identify CpG sites and differentially methylated regions (DMRs) in which DNAm levels in blood associated with lung function in Latino children and young adults with asthma. For that purpose, we performed several EWAS of pulmonary function test (PFT) measurements including forced expiratory volume in one second (FEV_1_), forced vital capacity (FVC), and their ratio (FEV_1_/FVC) pre- and post-administration of albuterol in Puerto Rican and Mexican American children and youth with asthma separately. Then, we assessed the ethnic-specific results for replication across the other ethnic subgroup. Moreover, we attempted to replicate in Latinos single epigenetic markers and DMRs associated with lung function in non-Latino populations.

## Results

### Characteristics of study populations

Characteristics of the 250 Puerto Ricans and 148 Mexican Americans from the Genes-Environment and Admixture in Latino Americans (GALA II) study included in the analysis are shown in Table 1. Overall, Puerto Ricans had lower pre- and post-FEV_1_% predicted and FVC % predicted than Mexican Americans (*p* < 0.05), although similar values were observed for the FEV_1_/FVC ratios. Among Puerto Ricans, the percentage of overweight participants was slightly lower than in Mexicans. Approximately, half of Puerto Ricans had normal weight and 27% were obese, while less than 40% of Mexican Americans had normal weight and approximately 40% were obese. In both subethnic groups, underweight individuals represented less than 10% of the populations. Exposure to second-hand smoking (SHS) and in utero maternal smoking was higher among individuals profiled with the Illumina Infinium HumanMethylation450 BeadChip array (450K) [17], compared with those where methylation was measured with the Illumina Infinium MethylationEPIC (EPIC).

**Table 1:** Characteristics of the GALA II study participants recruited between 2006 and 2014 that were included in the EWAS of lung function

Characteristic	EPIC		450 K	
	Puerto Rican	Mexican American	Puerto Rican	Mexican American
Sample size	160	42	90	106
Age (y)	13.3 ± 3.0	14.4 ± 3.8	12.0 ± 2.9	12.7 ± 3.8
Female *n* (%)	72 (45.0)	20 (47.6)	37 (41.1)†	63 (59.4)†
Height (cm)	154.3 ± 13.7	156.0 ± 14.4	150.2 ± 14.8	149.3 ± 14.7
Weight (Kg)	56.6 ± 20.3	63.8 ± 23.7	50.9 ± 23.6	57.5 ± 27.6
Body Mass Index (BMI) category, *n* (%)	*			
Underweight	3 (1.9)	0 (0)	7 (7.8)†	3 (2.8)†
Normal	81 (50.9)	15 (35.7)	52 (57.7)†	44 (41.5)†
Overweight	30 (18.9)	9 (21.4)	7 (7.8)†	14 (13.2)†
Obese	45 (28.3)	18 (42.9)	24 (26.7)†	45 (42.5)†
Pre-FEV_1_ (L)	2.4 (0.7)†	2.9 (0.9)†	2.3 (0.7)	2.5 (0.8)
Pre-FEV_1_% predicted	88.5 (14.7)†	98.9 (14.0)†	91.5 (12.8)†	99.2 (12.8)†
Pre-FVC (L)	2.8 (0.9)†	3.5 (1.2)†	2.7 (0.9)†	3.0 (1.0)†
Pre-FVC % predicted	90.2 (15.1)†	103.4 (13.8)†	93.7 (12.9)†	103.1 (12.6)†
Pre-FEV_1_/FVC (L)	0.86 (0.08)	0.84 (0.07)	0.86 (0.07)	0.85 (0.07)
Pre-FEV_1_/FVC % predicted	98.2 (8.9)†	96.0 (7.1)†	97.8(7.7)	96.4 (7.9)
Post-FEV_1_ (L)	2.7 (0.8)†	3.13 (0.9)†	2.5 (0.8)	2.65 (0.8)
Post-FEV_1_% predicted	98.1 (15.5)†	104.6 (12.3)†	99.8 (12.4)†	104.9 (12.5)†
Post-FVC (L)	3.0 (0.9)†	3.5 (1.0)†	2.9 (0.9)	3.0 (1.0)
Post-FVC % predicted	97.3 (15.8)†	105.1 (12.7)†	99.0 (13.8)†	105 (13.1)†
Post-FEV_1_/FVC (L)	0.88 (0.06)	0.87 (0.07)	0.88 (0.06)	0.88 (0.06)
Post-FEV_1_/FVC % predicted	100.6 (7.2)†	98.8 (6.3)†	100.9 (5.9)	100.3 (6.4)
In utero maternal smoking, *n* (%)	9 (5.7)	1 (2.4)	30 (33.3)†	13 (12.3)†
SHS, *n* (%)	30 (19.7)**	5 (11.9)	24 (26.7)	27 (26.7)**
Controller medication last 2 weeks, *n* (%)	34 (21.3)	13 (31.0)	25 (27.8)	39 (36.8)
Maternal high-school education, *n* (%)	139 (86.9)†	25 (59.5)†	68 (76.4)***†	67 (63.2)†
Insurance status, *n* (%)	†	****†	†	****†
Government	99 (61.9)	24 (60.0)	68 (75.6)	70 (66.7)
Private	57 (35.6)	10 (25.0)	22 (24.4)	28 (26.7)
None	4 (2.5)	6 (15.0)	0 (0)	7 (6.6)
Preterm birth (< 37 weeks), *n* (%)	56 (35.2)*****	12 (29.3)*****	30 (33.3)	26 (25.7)*****

^†^*P* value < 0.05 for the comparison of Mexicans and Puerto Ricans profiled with the same array

450K: Illumina Infinium HumanMethylation450 BeadChip 450K array; EPIC: Illumina Infinium MethylationEPIC array; FEV_1_: forced expiratory volume in 1 s; FVC: Forced vital capacity; Post: Post-administration of albuterol; Pre: Pre-administration of albuterol

^*^BMI was available for 159 Puerto Ricans profiled with the EPIC array. **SHS was available for 152 Puerto Ricans profiled with the EPIC array and 101 Mexicans profiled with the 450K array. ***Maternal education level was available for 89 Puerto Ricans profiled with the 450K array. ****Insurance status was available for 40 and 105 Mexicans profiled with the EPIC and 450K array, respectively. *****Preterm birth data was available for 159 Puerto Ricans and 41 Mexicans profiled with the EPIC array and 101 Mexicans profiled with the 450K array

For continuous variables, the mean and standard deviation are displayed, and the Mann–Whitney Wilcoxon test was applied for the comparison of cases versus controls. For categorical variables, the number and proportion of subjects in each category are shown and a *χ*^2^ test was applied for the comparison of cases versus controls

### Identification of CpGs associated with PFT measurements

**Fig. 1:**
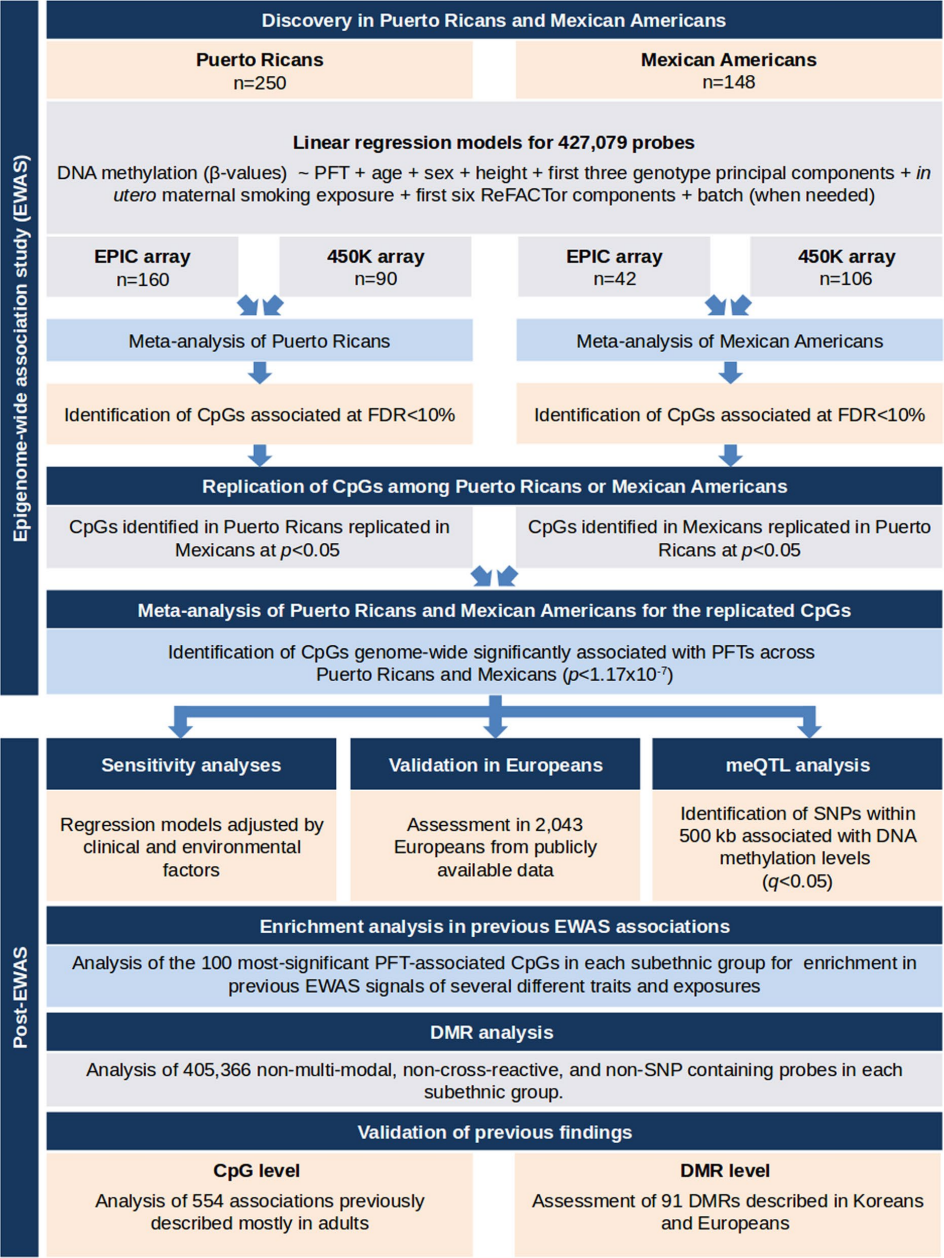
Study design of the EWAS of lung function in Mexican Americans and Puerto Ricans and post-EWAS analyses

We performed an EWAS of PFT in Mexican Americans and Puerto Ricans separately by DNA methylation array, meta-analyzed individuals within subethnic group, and performed replication between populations, as detailed in Fig. 1. A total of 18 CpGs showed suggestive association with PFTs at a false discovery rate (FDR)-adjusted *p* ≤ 0.1 in Puerto Ricans (Additional file 5: Table S1; Additional file 1: Fig. S1). In Mexican Americans, we found 132 CpGs that were suggestively associated with PFT measurements at FDR-adjusted *p* ≤ 0.1 (Additional file 5: Table S1; Additional file 2: Fig. S2). The quantile–quantile plots indicated no major signs of inflation (Additional file 3: Fig. S3).

From the CpGs identified in Puerto Ricans, the probes cg16405908 (*MRGPRE*, *p* = 1.64 × 10^−3^), cg07428101 (*MUC2*, *p* = 5.70 × 10^−3^) and cg00129273 (*PRDM14*, *p* = 1.17 × 10^−2^) had the same significant direction of the effect for post-FVC in Mexican Americans. From the CpGs associated with PFT in Mexican Americans, nine showed a significant (*p* < 0.05) and consistent direction of the effect (Table 2). The probes cg18573338 (*TBC1D17*) and cg18635207 (*TMEM90A*) were significantly associated with pre-FEV_1_ in Puerto Ricans (*p* = 4.64 × 10^−2^ and *p* = 4.37 × 10^−2^, respectively). One probe (cg10523319, *DHRS3*) showed association with pre-FVC in Puerto Ricans (*p* = 3.01 × 10^−2^). The probes cg00914963 (*TBC1D16*) and cg22467052 (*CFTR*) were associated with post-FVC in Puerto Ricans (*p* = 1.77 × 10^−2^ and *p* = 1.10 × 10^−2^, respectively). Likewise, the association of cg01408486 (*CXXC5)* and cg06035600 (*MAP3K6*) with pre-FEV_1_/FVC was replicated in Puerto Ricans (*p* = 2.45 × 10^−2^ and *p* = 3.69 × 10^−3^, respectively). The probes cg20515679 (*KCNJ6*) and cg25637972 (*RBM24*/*STMND1*) also exhibited a consistent significant association for post-FEV_1_/FVC in Puerto Ricans (*p* = 9.90 × 10^−4^ and *p* = 7.42 × 10^−3^, respectively).

**Table 2:** Association results for the probes that showed consistent association in Mexican Americans and Puerto Ricans from GALA II

Trait	Discovery/replication	Probe	Gene	Discovery				Replication			Meta-analysis		
				Coef	SE	*P*	FDR	Coef	SE	*P*	Coef	SE	*P*
Post-FVC	PR/MX	cg16405908	*MRGPRE*	− 2.53 × 10^−2^	5.05 × 10^−3^	5.13 × 10^−7^	0.071	− 1.79 × 10^−2^	5.70 × 10^−3^	1.64 × 10^−3^	− 2.21 × 10^−2^	3.78 × 10^−3^	**5.02 × 10**^−**9**^
Post-FVC	PR/MX	cg07428101	*MUC2*	7.21 × 10^−3^	1.47 × 10^−3^	8.72 × 10^−7^	0.071	5.64 × 10^−3^	2.04 × 10^−3^	5.70 × 10^−3^	6.68 × 10^−3^	1.19 × 10^−3^	**2.05 × 10**^−**8**^
Post-FVC	PR/MX	cg00129273	*PRDM14*	− 5.86 × 10^−2^	5.28 × 10^−2^	1.16 × 10^−6^	0.071	− 4.27 × 10^−2^	1.69 × 10^−2^	1.17 × 10^−2^	− 5.24 × 10^−2^	2.59 × 10^−2^	1.80 × 10^−7^
Pre-FEV1	MX/PR	cg18573338	*TBC1D17*	8.71 × 10^−3^	8.42 × 10^−3^	7.38 × 10^−7^	0.061	2.62 × 10^−3^	1.32 × 10^−3^	4.64 × 10^−2^	5.32 × 10^−3^	2.97 × 10^−3^	2.20 × 10^−6^
Pre-FEV1	MX/PR	cg18635207	*TMEM90A*	4.22 × 10^−3^	4.04 × 10^−3^	8.56 × 10^−7^	0.061	1.17 × 10^−3^	5.78 × 10^−4^	4.37 × 10^−2^	2.45 × 10^−3^	1.35 × 10^−3^	2.32 × 10^−6^
Pre-FVC	MX/PR	cg10523319	*DHRS3*	6.55 × 10^−3^	1.25 × 10^−3^	1.70 × 10^−7^	0.012	2.38 × 10^−3^	3.22 × 10^−3^	3.01 × 10^−2^	4.70 × 10^−3^	2.10 × 10^−3^	1.31 × 10^−7^
Pre-FEV1/FVC	MX/PR	cg06035600	*MAP3K6*	3.35 × 10^−1^	6.89 × 10^−2^	1.15 × 10^−6^	0.068	1.81 × 10^−1^	6.22 × 10^−2^	3.69 × 10^−3^	2.50 × 10^−1^	4.62 × 10^−2^	**6.13 × 10**^−**8**^
Pre-FEV1/FVC	MX/PR	cg01408486	*CXXC5*	1.12 × 10^−1^	2.42 × 10^−2^	3.51 × 10^−6^	0.068	4.79 × 10^−2^	2.13 × 10^−2^	2.45 × 10^−2^	7.60 × 10^−2^	1.60 × 10^−2^	2.02 × 10^−6^
Post-FVC	MX/PR	cg00914963	*TBC1D16*	− 2.53 × 10^−2^	4.96 × 10^−3^	3.33 × 10^−7^	0.016	− 1.08 × 10^−2^	4.55 × 10^−3^	1.77 × 10^−2^	− 1.89 × 10^−2^	5.76 × 10^−3^	**1.04 × 10**^−**7**^
Post-FVC	MX/PR	cg22467052	*CFTR*	8.01 × 10^−3^	6.09 × 10^−3^	2.15 × 10^−6^	0.044	3.44 × 10^−3^	1.35 × 10^−3^	1.10 × 10^−2^	5.76 × 10^−3^	2.55 × 10^−3^	6.03 × 10^−7^
Post-FEV1/FVC	MX/PR	cg20515679	*KCNJ6*	− 1.86 × 10^−1^	1.35 × 10^−1^	2.58 × 10^−6^	0.046	− 8.53 × 10^−2^	2.59 × 10^−2^	9.90 × 10^−4^	− 1.24 × 10^−1^	4.84 × 10^−2^	**1.13 × 10**^−**7**^
Post-FEV1/FVC	MX/PR	cg25637972	*RBM24/STMND1*	7.28 × 10^−2^	1.56 × 10^−2^	3.13 × 10^−6^	0.051	4.37 × 10^−2^	1.63 × 10^−2^	7.42 × 10^−3^	5.89 × 10^−2^	1.13 × 10^−2^	1.79 × 10^−7^

Coef: Regression coefficient estimate; FEV_1_: forced expiratory volume in 1 s; FDR: False discovery rate adjusted *p* value within each phenotype; FVC: Forced vital capacity; MX: Mexicans; *P*: *P* value; PR: Puerto Ricans; SE: Standard error of the regression coefficient estimate. Genome-wide significant association *p* values after meta-analysis are in boldface

**Fig. 2:**
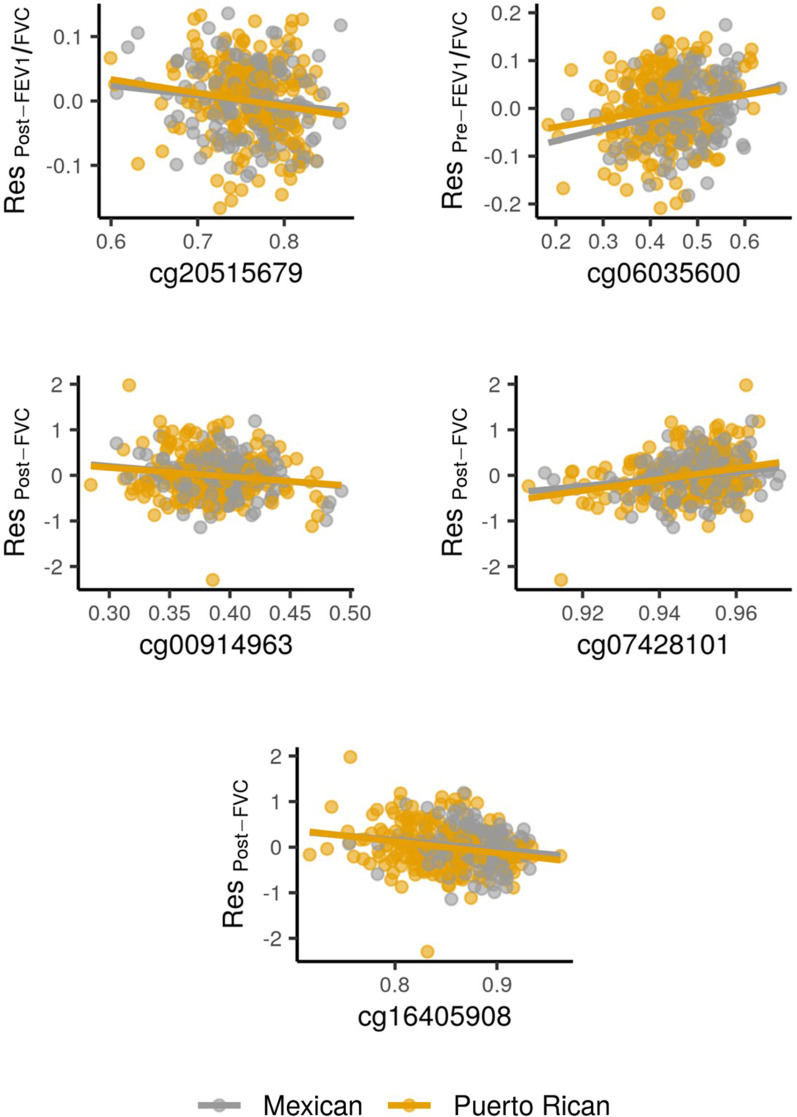
Correlation between lung function measurements and DNA methylation levels at the five genome-wide significant CpG sites in the meta-analysis of Puerto Ricans and Mexican Americans. The DNA methylation levels are shown as beta-values in the *x*-axis along the residuals of the regression of the lung function measurement adjusted by the covariates age, sex, height, in utero smoking exposure and genetic ancestry (represented in the *y*-axis)

From the probes that were significant in the discovery stage and replicated across ethnic sub-groups, five exceeded the genome-wide significance threshold for significance based on the number of probes analyzed (*p* = 0.05/427,079 = 1.17 × 10^−7^) in the meta-analysis of Mexican Americans and Puerto Ricans. These genome-wide significant hits included cg06035600 (*MAP3K6*, *p* = 6.13 × 10^−8^) for pre-FEV_1_/FVC, cg20515679 (*KCNJ6*, *p* = 1.13 × 10^−7^) for post-FEV_1_/FVC, and the probes cg00914963 (*TBC1D16*, *p* = 1.04 × 10^−7^), cg16405908 (*MRGPRE*, *p* = 5.02 × 10^−9^), and cg07428101 (*MUC2*, *p* = 2.05 × 10^−8^) for post-FVC (Fig. 2). None of these five genome-wide significant probes was flagged as cross-reactive, multi-modal, or single nucleotide polymorphism (SNP)-containing probes (not flagged in Additional file 5: Table S1).

### Sensitivity analyses accounting for potential confounders

We next performed sensitivity analyses including body mass index (BMI) category, SHS exposure, use of asthma controller medication in the two weeks preceding the spirometry, insurance status, and maternal education level. However, minimal differences in the effect sizes for the five genome-wide significant CpG sites were detected (Table 3), which suggests no major effect of these factors. We also explored whether the effects were consistent across the two arrays used for methylation profiling and similar effects were found (Additional file 5: Table S2).

**Table 3:** Sensitivity analysis for the genome-wide significant hits in the meta-analysis across both stages

Probe	*Model*	Puerto Ricans				Mexicans				Meta-analysis			
		Coef	SE	*P*	CQP	Coef	SE	*P*	CQP	Coef	SE	*P*	CQP
cg16405908	M0	− 2.53E−02	5.05E−03	5.13E−07	2.48E−01	− 1.79E−02	5.70E−03	1.64E−03	7.87E−01	− 2.21E−02	3.78E−03	**5.02E**−**09**	5.02E−01
	M1	− 1.75E−02	5.99E−03	3.40E−03	8.09E−01	− 2.45E−02	5.18E−03	2.33E−06	2.98E−01	− 2.15E−02	3.92E−03	**4.06E−08**	5.93E−01
	M2	− 1.70E-02	5.75E−03	3.17E−03	9.94E−01	− 2.63E−02	5.35E−03	8.54E−07	2.99E−01	− 2.20E−02	3.92E−03	**1.97E−08**	4.76E−01
	M3	− 1.79E-02	5.77E−03	1.93E−03	8.63E−01	− 2.54E−02	5.08E−03	5.69E−07	2.54E−01	− 2.21E−02	3.81E−03	**6.50E−09**	5.16E−01
	M4	− 1.83E-02	6.02E−03	2.33E−03	9.32E−01	− 2.49E−02	5.06E−03	8.73E−07	2.36E−01	− 2.22E−02	3.87E−03	**1.04E−08**	5.50E−01
	M5	− 1.77E-02	5.78E−03	2.15E−03	7.22E−01	− 2.57E−02	5.04E−03	3.55E−07	2.41E−01	− 2.22E−02	3.80E−03	**4.77E−09**	4.64E−01
	M6	− 1.86E-02	5.74E−03	1.22E−03	6.95E−01	− 2.50E−02	5.10E−03	9.14E−07	2.21E−01	− 2.22E−02	3.81E−03	**5.95E−09**	5.01E−01
	M7	− 1.87E-02	6.46E−03	3.86E−03	8.96E−01	− 2.36E−02	5.66E−03	3.03E−05	2.11E−01	− 2.15E−02	4.26E−03	4.62E−07	5.90E−01
cg07428101	M0	7.21E-03	1.47E−03	8.72E−07	6.55E−01	5.64E−03	2.04E−03	5.70E−03	4.64E−01	6.68E−03	1.19E−03	**2.05E−08**	7.71E−01
	M1	5.65E-03	2.15E−03	8.61E−03	6.04E−01	6.91E−03	1.50E−03	3.93E−06	5.84E−01	6.50E−03	1.23E−03	1.23E−07	8.50E−01
	M2	4.62E-03	1.91E−03	1.57E−02	6.79E−01	7.13E−03	1.57E−03	5.93E−06	6.70E−01	6.11E−03	1.22E−03	4.87E−07	7.10E−01
	M3	5.88E-03	2.07E−03	4.58E−03	4.20E−01	7.24E−03	1.47E−03	9.03E−07	6.66E−01	6.78E−03	1.20E−03	**1.64E−08**	7.72E−01
	M4	5.51E-03	2.22E−03	1.32E−02	3.45E−01	7.29E−03	1.48E−03	8.49E−07	6.69E−01	6.75E−03	1.23E−03	**4.44E−08**	6.78E−01
	M5	5.18E-03	2.05E−03	1.14E−02	2.86E−01	7.24E−03	1.47E−03	8.04E−07	7.14E−01	6.54E−03	1.19E−03	**4.14E−08**	5.84E−01
	M6	5.81E-03	2.12E−03	6.10E−03	6.48E−01	7.02E−03	1.49E−03	2.60E−06	6.11E−01	6.62E−03	1.22E−03	**5.89E−08**	8.77E−01
	M7	4.68E-03	2.22E−03	3.49E−02	5.99E−01	6.36E−03	1.69E−03	1.71E−04	5.85E−01	5.74E−03	1.35E−03	1.98E−05	8.16E−01
cg06035600	M0	3.35E-01	6.89E−02	1.15E−06	6.11E−01	1.81E−01	6.22E−02	3.69E−03	8.84E−01	2.50E−01	4.62E−02	**6.13E−08**	3.83E−01
	M1	3.38E-01	7.29E−02	3.51E−06	5.64E−01	1.78E−01	6.23E−02	4.31E−03	8.72E−01	2.46E−01	4.74E−02	2.17E−07	3.70E−01
	M2	3.51E-01	7.23E−02	1.24E−06	7.26E−01	1.57E−01	6.52E−02	1.59E−02	7.88E−01	2.44E−01	4.84E−02	4.72E−07	2.46E−01
	M3	3.34E-01	6.91E−02	1.30E−06	5.08E−01	1.64E−01	6.25E−02	8.63E−03	8.73E−01	2.41E−01	4.63E−02	2.05E−07	2.83E−01
	M4	3.06E-01	7.24E−02	2.45E−05	9.92E−01	1.76E−01	6.18E−02	4.30E−03	9.31E−01	2.31E−01	4.70E−02	9.08E−07	6.05E−01
	M5	3.38E-01	6.99E−02	1.35E−06	5.80E−01	1.93E−01	6.26E−02	2.05E−03	9.23E−01	2.58E−01	4.67E−02	**3.39E−08**	4.41E−01
	M6	3.02E-01	7.02E−02	1.68E−05	8.44E−01	1.79E−01	6.27E−02	4.30E−03	8.77E−01	2.34E−01	4.68E−02	5.86E−07	6.20E−01
	M7	3.09E-01	8.78E−02	4.41E−04	8.14E−01	1.51E−01	6.62E−02	2.21E−02	7.30E−01	2.08E−01	5.29E−02	8.05E−05	5.29E−01
cg00914963	M0	− 2.53E-02	4.96E−03	3.33E−07	6.70E−01	− 1.08E−02	4.55E−03	1.77E−02	7.24E−02	− 1.89E−02	5.76E−03	**1.04E−07**	4.50E−02
	M1	− 2.70E-02	5.13E−03	1.47E−07	9.72E−01	− 9.98E−03	4.72E−03	3.44E−02	6.19E−02	− 1.99E−02	6.44E−03	**1.10E−07**	2.41E−02
	M2	− 2.49E-02	4.97E−03	5.51E−07	2.69E−01	− 1.17E−02	1.19E−02	5.66E−02	2.09E−02	− 1.73E−02	7.33E−03	3.77E−07	5.02E−03
	M3	− 2.46E-02	4.98E−03	7.73E−07	6.30E−01	− 1.04E−02	4.55E−03	2.18E−02	6.74E−02	− 1.84E−02	5.74E−03	2.66E−07	4.62E−02
	M4	− 2.92E-02	4.77E−03	9.43E−10	8.75E−01	− 1.16E−02	4.51E−03	9.92E−03	9.80E−02	− 2.15E−02	6.18E−03	**4.06E−10**	1.91E−02
	M5	− 2.57E-02	5.03E−03	3.30E−07	7.70E−01	− 1.06E−02	4.55E−03	2.03E−02	9.03E−02	− 1.88E−02	5.76E−03	1.44E−07	4.77E−02
	M6	− 2.31E-02	5.09E−03	5.63E−06	2.79E−01	− 1.08E−02	4.64E−03	2.05E−02	6.91E−02	− 1.64E−02	3.43E−03	1.83E−06	5.28E−02
	M7	− 2.94E-02	5.40E−03	5.16E−08	4.47E−01	− 1.05E−02	1.30E−02	9.29E−02	1.64E−02	− 1.89E−02	8.90E−03	**1.01E−07**	1.01E−03
cg20515679	M0	− 1.86E-01	1.35E−01	2.58E−06	3.54E−04	− 8.53E−02	2.59E−02	9.90E−04	4.04E−01	− 1.24E−01	4.84E−02	**1.13E−07**	1.94E−03
	M1	− 1.83E-01	1.35E−01	2.78E−06	4.47E−04	− 8.96E−02	2.65E−02	7.13E−04	5.04E−01	− 1.26E−01	4.91E−02	**8.80E−08**	2.55E−03
	M2	− 1.85E-01	1.36E−01	5.02E−06	3.84E−04	− 8.76E−02	2.63E−02	8.67E−04	4.05E−01	− 1.24E−01	4.83E−02	1.79E−07	2.42E−03
	M3	− 1.95E-01	1.31E−01	5.58E−07	4.10E−04	− 8.32E−02	2.60E−02	1.37E−03	4.81E−01	− 1.28E−01	4.89E−02	**3.89E−08**	1.60E−03
	M4	− 2.14E-01	1.59E−01	8.13E−06	2.90E−04	− 8.15E−02	2.60E−02	1.68E−03	4.47E−01	− 1.28E−01	5.10E−02	8.87E−07	1.75E−03
	M5	− 1.94E-01	1.42E−01	1.24E−06	2.05E−04	− 8.51E−02	2.61E−02	1.12E−03	4.23E−01	− 1.27E−01	5.06E−02	**7.13E−08**	1.12E−03
	M6	− 1.97E-01	1.40E−01	7.92E−06	5.76E−04	− 8.42E−02	2.58E−02	1.12E−03	4.27E−01	− 1.25E−01	4.78E−02	4.34E−07	3.13E−03
	M7	− 2.21E-01	1.44E−01	7.86E−05	4.00E−03	− 8.46E−02	2.70E−02	1.73E−03	6.83E−01	− 1.27E−01	4.52E−02	6.22E−06	1.82E−02

BMI: BMI category; CM2: Use of controller medication in the 2 weeks preceding spirometry; CQP: *P* value of Cochran’s Q; Coef: Regression coefficient estimate; FEV_1_: forced expiratory volume in 1 s; FVC: Forced vital capacity; SHS: Second-hand smoking exposure; *P*: *P* value; SE: Standard error of the regression coefficient estimate. Genome-wide significant *p* values are shown in bold

M0: CpG ~ PFT + Age + Sex + Height + In utero maternal smoking exposure + Genetic ancestry + Cellular heterogeneity + Batch; M1: CpG ~ M0 + BMI; M2: CpG ~ M0 + SHS; M3: CpG ~ M0 + Controller medication use in the past two weeks; M4: CpG ~ M0 + insurance status; M5: CpG ~ M0 + maternal education level; M6: CpG ~ M0 + Preterm birth; M7: CpG ~ M0 + BMI + SHS + Controller medication use in the past two weeks + insurance status + maternal education level + preterm birth

We next tested the association of the genome-wide significant probes with the exposure to air pollution during the past year and lifetime, including daily average of 1-h ozone (O_3_), sulfur dioxide (SO_2_), nitrogen dioxide (NO_2_), particulate matter ≤ 10 µm in diameter (PM_10_) or 2.5 µm (PM_2.5_) (Additional file 5: Table S3). The probe cg07428101 exhibited association at *p* < 0.05 with lifetime daily average of 1-h O_3_ (*p* = 1.77 × 10^−2^). Moreover, the probe cg00914963 showed significant association with PM2.5 and O_3_ exposure in the past year at *p* < 0.05 (*p* = 6.94 × 10^−3^ and *p* = 2.18 × 10^−2^, respectively), and with the participant’s lifetime exposure (*p* = 1.16 × 10^−2^ and *p* = 2.32 × 10^−2^, respectively). However, the association with PFT measurements was not confounded by air pollution exposure (Additional file 5: Table S4).

### Assessment of genome-wide significant association hits for replication in Europeans

The replication in non-Latino populations of the 5 CpG sites that showed genome-wide significant association in the meta-analysis of Latinos was assessed in 2,043 European adults, including all individuals and a subset of never smokers. However, none of these were associated with PFT in publicly available data from European adults [18] (Additional file 5: Table S5).

### Methylation quantitative trait loci analysis

A cis-methylation quantitative trait loci (meQTL) analysis was performed to test whether genetic variation was associated with DNAm levels at the associated CpGs. The five tested CpG sites were genetically regulated. From the 13,668 SNPs evaluated, 785 meQTLs exhibited association at Storey *q*-value < 0.05 (Additional file 5: Table S6). A total of 78 quasi-independent SNPs identified by linkage disequilibrium clumping of SNPs with pairwise *r*^2^ < 0.25 within 250 kilobases. These were distributed per CpG site as follows: cg00914963 (14 SNPs), cg06035600 (10 SNPs), cg07428101 (11 SNPs), cg16405908 (40 SNPs), and cg20515679 (3 SNPs). From these, 16 were replicated in the Biobank-based Integrative Omics Studies (BIOS) consortium [19] (FDR < 0.05) (Additional file 5: Table S7). From the 78 meQTLs, the SNPs rs61870478 (*p* = 3.01 × 10^−3^), rs74382103 (*p* = 2.69 × 10^−2^), rs2362396 (*p* = 3.51 × 10^−2^), and rs234850 (*p* = 4.60 × 10^−2^) showed association with post-FVC (%) predicted at *p* < 0.05 among Latinos in an analysis adjusted by age, sex, and the first three genotype principal components (Additional file 5: Table S8). As post-FVC is not available in the Pan-UK Biobank [20], we tested the association with pre-FVC-associated traits, but no meQTL was replicated (*p* > 0.05) (Additional file 5: Table S9).

### Enrichment analyses in previous EWAS signals

Among the 100 most significant PFT associated probes for each subethnic group, there was a significant enrichment in previous EWAS signals for known factors involved in lung function, including respiratory diseases (asthma and COPD), allergic phenotypes (respiratory allergies and allergic sensitization), BMI, pollutants exposure, smoking, socioeconomic status, alcohol consumption, ancestry, or circadian rhythm (Additional file 4: Fig. S4). Additionally, enrichment in associations for traits related to autoimmune diseases, mortality, and preterm birth were also shared among several PFTs.

### Identification of differentially methylated regions associated with PFT measurements

We next assessed lung function-related DMRs (Additional file 5: Table S10) for a total of 405,366 non-multi-modal, non-cross-reactive and non-SNP-containing CpG sites, as detailed in Additional file 6. Multiple DMRs in genes involved in airway remodeling or inflammation overlapped across phenotypes, such as pre- and post-FEV_1_ as well as pre- and post-FVC in Mexican Americans (e.g., *TNFRSF14/HES5, MAN2B1/ZNF791,* and *MRPS23/VEZF1*) and Puerto Ricans (e.g., *CLMN/SYNE3,* and *GSDMD*). Moreover, two regions that contain 3 CpGs (*REXO1*) and 8 CpGs (*AURKC*) were associated with pre-FEV_1_/FVC in both subethnic groups.

### Replication of previous epigenetic loci for lung function

The analyses in Mexican Americans showed significant enrichment in previous PFT-associated epigenetic loci [11, 18, 21–28] for pre-FEV_1_ (*p* = 0.031), pre-FEV_1_/FVC (*p* = 0.009), and post-FEV_1_ (*p* = 0.006), whereas Puerto Ricans showed significant enrichment for pre-FEV_1_/FVC (*p* = 0.002) (Additional file 5: Table S11). Previous PFT-associated CpGs that were associated in either subethnic group at nominal level (*p* < 0.05) are shown in Additional file 5: Table S12. Among those probes that showed significant association in both subethnic groups separately, 19 probes also had *p* < 0.05 in the combined results of both subethnic groups, and four of these were associated with multiple PFT traits: cg16734845 (*CTDSPL2*), cg25634666 (*FOLR3*), cg07148038 (*TNXB*), and cg26206598 (*PREX1*). However, none exceeded the Bonferroni-corrected threshold, accounting for the number of probes assessed for replication (*p* = 0.05/554 tested probes = 9.02 × 10^−5^). Regarding the DMRs, a total of three out of the 54 DMRs previously associated with PFTs in Korean adults [11] showed Šidák-corrected *p* < 0.05 in Mexican Americans, but not in Puerto Ricans. Specifically, the DMR at the *BHMT* region associated with pre- and post-FEV_1_, the DMR at *F2R *was associated with pre- and post-FEV_1_/FVC, and the DMR at *TACR3* was associated with post-FEV_1_/FVC. Additionally, a DMR at *ZNF429* was associated with pre-FEV_1_, although the region limits differed from those reported previously for a DMR of FEV_1_/FVC [11]. However, one of the 37 DMRs from cord blood previously associated with childhood lung function in Europeans [18] was replicated in Puerto Ricans (Šidák-corrected *p* > 0.05).The DMR at this region was significantly associated with Pre- and Post-FVC and Post-FEV_1_.

## Discussion

To our knowledge, this is the first EWAS of lung function in whole blood from Puerto Rican and Mexican American children and young adults with asthma. We identified five differentially methylated probes that showed genome-wide significant association with lung function, including one CpG site with evidence of being regulated by genetic variation. DNA methylation at these probes was genetically regulated according to the meQTL analysis. We also flagged two DMRs associated with lung function that were shared among both Puerto Ricans and Mexican Americans. Moreover, we validated five DMRs in Latinos (four were previously reported in Koreans and one in Europeans) and several CpG sites originally reported in European adults.

In the EWAS, we set a looser threshold for suggestive significance, with an FDR-adjusted *p* < 0.10, in order to identify potentially relevant sites for replication and established a stringent Bonferroni-corrected genome-wide significance threshold significance to control for false positives in the combined analysis of Mexican Americans and Puerto Ricans. This led to the identification of two CpG sites associated in Puerto Ricans that replicated in Mexican Americans and three CpGs associated in Mexican Americans that replicated in Puerto Ricans.

The two CpG sites discovered in Puerto Ricans were associated with post-FVC and annotated to genes that play a role in mucosal tissues. The probe cg16405908 was annotated to *MRGPRE*, which is expressed in whole-blood and lung [29]. Despite the fact that the role of *MRGPRE* is unknown, Mas-related G protein coupled receptors are involved in nociception homeostasis, bronchoconstriction, and airway hyperresponsiveness [30]. The other probe (cg07428101) was located in the intronic region of *MUC2*, a mucin expressed in the airway mucosa. In contrast to the genes encoding for other mucins (i.e., *MUC5AC* and *MUC5B*) located nearby *MUC2*, with an important role on mucus homeostasis and airway inflammation, the role of *MUC2* is less known [31]. Still, it is plausible that DNA methylation levels at this CpG may exert regulatory effects over genes nearby. Another post-FVC-associated probe was cg00914963, annotated to *TBC1D16*, which encodes a RAB GTPase that promotes GTP hydrolysis by Rab4A, which in turn mediates *VEGFR2* trafficking in endothelial cells and thereby regulates vascular permeability [32]. Interestingly, *VEGFR2* is overexpressed in pulmonary tissue from patients with COPD and it is related to disease severity [29]. Indeed, among COPD patients, *VEGFR2* expression in lung is negatively correlated with lung function [33].

The probe cg06035600 (*MAP3K6*) was associated with post-FEV_1_/FVC and has been previously related to aging and smoking [34]. However, the effect of the association was not modified by passive smoke exposure in our analyses. Reduced expression of *MAP3K6* downregulates *VEGF*, which alters normal angiogenesis [35]. Moreover, *MAP3K6* participates in cell signaling and subsequent regulation of gene expression [36].

The cg20515679 probe from *KCNJ6*, which was associated with post-FEV_1_/FVC, has also been associated with Crohn's disease and NO_2_ according to EWASatlas [34]. However, the association with NO_2_ described previously in an adult Dutch population-based cohort [37] was not replicated in our data. Moreover, *KCNJ6* is also upregulated in mild and severe asthma in peripheral blood cells [38]. Although genetic variation in *KCNJ6* has been associated with FVC [39], we did not find any significant SNP-CpG pairs in Latinos or Europeans.

The lack of replication of the genome-wide significant CpGs in European adults could be due to differences in age, population background, study design, and analytical methods. The association of reduced lung function and African ancestry among Puerto Ricans [14, 15] and the significant enrichment in ancestry-associated probes for several PFTs (Additional file 4: Fig. S4) suggests the existence of population-specific drivers among the Latino population. Nevertheless, several previously reported probes were associated at *p* < 0.05 in both subethnic groups.

The two DMRs consistently associated in Puerto Ricans and Mexican Americans were annotated to genes that were not known to be implicated in PFTs, but that are likely implicated in inflammatory or regulatory processes. *AURKC* encodes a serine/threonine kinase involved in histone phosphorylation and cell division, whose expression is induced by tumor necrosis factor-alpha in response to the inflammation-related transcription factor CEBPD [40]. Little is known about the protein encoded by the *REXO1* gene, other than its participation in RNA polymerase II transcription via Elongin A [41] and its role promoting cervical cancer cell proliferation and progression [42].

It is worth noting that the association signals identified here were enriched in biologically relevant diseases and traits, including chronic inflammatory diseases and preterm birth. Interestingly, prematurity is inversely associated with airflow limitation in children [43] and adults [44]. However, despite DNAm may have a role in the interplay of gestational age and lung function in pediatric asthma, preterm birth did not explain the association of the identified CpG sites with PFTs.

Some limitations of this study must be acknowledged. First, the sample size of our study is modest in comparison to previous EWAS in individuals of European descent, especially to detect subethnic-specific in Mexican Americans and Puerto Ricans separately. Second, long-term longitudinal methylation changes could not be evaluated due to the cross-sectional design of GALA II. Third, since measured cell counts were not available, we used a reference-free method to adjust for effects of cell-type heterogeneity on DNAm patterns. Fourth, the fact that we analyzed the markers that were shared between two different arrays, one of them with a reduced number of markers, limited our genomic coverage. Despite these limitations, our study was strengthened by the fact that first, we focused on methylation profiling of whole blood from minority populations understudied in previous EWAS of PFTs. Second, we conducted both single-marker and region-based analyses and we evaluated whether genome-wide significant epigenetic loci were genetically regulated in Hispanics/Latinos with asthma. Third, we assessed several potential confounders on the association with epigenetic loci, including prematurity, BMI, in utero and current SHS exposure, medication use, air pollution exposure, and socioeconomic factors (insurance status and maternal education level).

## Conclusions

In summary, we identified consistent DNA methylation patterns in whole blood associated with lung function in pediatric asthma among Mexican Americans and Puerto Ricans that may be population-specific for Latinos/Hispanics. Moreover, we replicated previous findings originally described in non-Latino/Hispanic populations. These results provide insights into the mechanisms involved in lung function.

## Methods

### Study participants

GALA II is a case–control study of pediatric asthma in Hispanics/Latinos that were recruited between 2006 and 2014 in five areas from the US (Chicago, Bronx, Houston, San Francisco Bay Area) and Puerto Rico (San Juan) [45]. Briefly, individuals were included if they were aged between 8 and 21 years old, self-identified as Hispanic or Latino, and had four Latino grandparents. Asthma was defined by physician diagnosis, use of controller or rescue medication, and report of two or more symptoms of coughing, wheezing, or shortness of breath. Exclusion criteria for the study included the following: (1) any smoking within one year of the recruitment date; (2) 10 or more pack-years of smoking; (3) pregnancy in the third trimester; (4) history of lung diseases other than chronic illness.

### Pulmonary function tests

Pre- and post-bronchodilation spirometric data for FEV_1_, FVC, and FEV_1_/FVC were recorded with a KoKo® PFT Spirometer (nSpire Health Inc., Louisville, CO) according to the American Thoracic Society recommendations [46]. Post-bronchodilator PFT measurements were assessed 15 min after providing the participants a dose of albuterol, consisting of 4 puffs of albuterol (360 μg). Raw values were normalized as predicted percentages based on predicted values from the Global Lung Initiative 2012 (GLI-12) reference equation [47].

### Methylation profiling and quality control

DNAm measurements were obtained from whole blood using the Infinium EPIC BeadChip or the Infinium HumanMethylation450 BeadChip array (Illumina, San Diego, CA, USA). Methylation profiling and quality control (QC) are detailed in Additional file 6. QC was performed with the ENmix (1.22.0) R package [48] (Additional file 5: Table S13). Low quality probes (beads < 3 or a detection *p* value > 1 × 10^−6^ for ≥ 5% of the samples) and samples with low quality data points for ≥ 5% of the CpG sites were removed along with samples with a total bisulfite intensity less than 3 standard deviations of the sample bisulfite control, outliers of the total bisulfite intensity or beta value distribution. We then performed background correction, dye-bias correction, inter-array normalization, and probe-type bias adjustment. Missing values were imputed after removal of samples with more than 10% of missing probes as well as probes with missing values in more than 5% of the samples. Samples with mismatched sex or mixed genotype distributions on the control SNP probes and probes on sex chromosomes were excluded. After QC, 427,079 probes that overlapped in both arrays were available for subsequent analysis.

### Whole-genome sequencing

WGS was performed at the New York Genome Center and Northwest Genomics Center on an Illumina HiSeq X system. DNA processing, quality control, library construction, WGS, read processing, and sequence data quality control are described elsewhere [49]. Genotypes used in this study were based on TOPMed freeze 8 data with a minimal depth of 10 (DP10).

### Statistical analysis

Genetic ancestry assessment was performed by a principal component (PC) analysis of genotype data, as detailed in Additional file 6. Cell-type heterogeneity was captured using ReFACTor [50] within the GLINT [51] v1.09 framework. The association of DNAm beta-values and raw PFT values (in liters) was tested by robust linear regressions with correction for age, sex, height, the first three genotype principal components (PCs), in utero maternal smoking exposure, the first six ReFACTor components, and batch, when appropriate, via *limma* R package [52]. Meta-analysis of fixed- or random-effects models, based on Cochran's Q *p* value, was conducted with METASOFT [53]. In silico replication of the significant probes in Europeans was performed using publicly available data [18].

Prior to downstream analysis, we removed previously reported cross-reactive probes, probes with a SNP at a single base extension (SBE) or at the CpG site with minor allele frequency (MAF) > 0.01, and multimodal probes.

Beta values, ranging from 0 to 1, were transformed to M-values as log_2_(*β*/(1 − *β*)) for downstream analysis. To assess the effects of genetic variation on DNA methylation, we conducted meQTL analysis using fastQTL [54]. Genetic variants located ± 500 kilobases of the probe site and with MAF ≥ 0.01 in at least 10 samples were considered. For each subethnic group and array, linear regression models were corrected for age, sex, genotype PCs, in utero maternal smoking exposure, ReFACTor components, and batch, when appropriate. Moreover blood meQTLs were assessed in the Biobank-based Integrative Omics Studies (BIOS) consortium data [19].

Additionally, we tested for enrichment in previous EWAS signals for the top 100 probes using EWAS toolkit [34] and enrichment in previous PFT signals in the full EWAS using Fisher’s exact test. DMRs were assessed using the uncorrected *p* values with comb-p [55]. Moreover, we assessed for replication CpG sites and DMRs previously associated with lung function. Further details are described in Additional file 6.

## Supplementary Information

**Additional file 1: Fig. S1**. Manhattan-plot for the EWAS of lung function in Puerto Ricans. (A) pre-forced expiratory volume in 1 s (FEV_1_), (B) pre-forced vital capacity (FVC), (C) pre-FEV_1_/FVC ratio, (D) post-FEV_1_, (E) post-FVC, and (F) post-FEV_1_/FVC ratio. The statistical significance of association results (− log_10_ *p* value) is represented for each CpG site as a dot (*y*-axis) along the autosomal chromosomes (*x*-axis) from chromosome 1 (left) to chromosome 22 (right). The threshold for a false discovery rate less than 1% for each trait of lung function is indicated by the dashed gray line and the genome-wide threshold for significance is represented by the continuous gray line.

**Additional file 2: Fig. S2**. Manhattan-plot for the EWAS of lung function in Mexican Americans. (A) pre-forced expiratory volume in 1 s (FEV_1_), (B) pre-forced vital capacity (FVC), (C) pre-FEV_1_/FVC ratio, (D) post-FEV_1_, (E) post-FVC, and (F) post-FEV_1_/FVC ratio. The statistical significance of association results (− log_10_ *p* value) is represented for each CpG site as a dot (*y*-axis) along the autosomal chromosomes (*x*-axis) from chromosome 1 (left) to chromosome 22 (right). The threshold for a false discovery rate less than 1% for each trait of lung function is indicated by the dashed gray line and the genome-wide threshold for significance is represented by the continuous gray line.

**Additional file 3: Fig. S3**. Quantile–quantile plot for the EWAS of the association between each specific lung function measurement and DNA methylation in Mexican Americans (MEX) and Puerto Ricans (PR). For the EWAS in Mexican Americans, inflation factors were 1.02, 1.13, 1.24, 1.03, 1.12 and 1.24 for pre-forced expiratory volume in 1 s (FEV_1_), pre-forced vital capacity (FVC) and their pre-ratio (FEV1/FVC) and post-FEV_1_, post-FVC and post-FEV_1_/FVC, respectively. For the EWAS in Puerto Ricans, inflation factors were 1.19, 1.17, 1.09, 1.22, 1.21, 1.20 for pre-FEV1, pre-FVC, pre-FEV1/FVC, post-FEV_1_, post-FVC and post-FEV_1_/FVC, respectively. The observed *p* value (− log_10_ *p* value) is shown in the *y*-axis along the expected *p* value (− log_10_ *p* value) represented in the *x*-axis.

**Additional file 4: Fig. S4**. Heatmap of the trait enrichment for the top 100 CpGs for each pulmonary function test and ethnicity. Significant results at an FDR < 0.05 for forced expiratory volume in 1 s (Pre-FEV_1_), forced vital capacity (FVC) and their ratio (FF) pre- and post-administration of albuterol are shown for Puerto Ricans (PR) and Mexican Americans (MX). The enrichment raw *p* value is colored on a scale from blue (less significant association) to red (more significant association). Non-significant *p* values are represented in gray.

**Additional file 5.** Supplementary Tables S1-S13.

**Additional file 6.** Supplementary Material.

## Data Availability

Whole-genome sequencing is available at the database of Genotypes and Phenotypes (dbGaP) (Study Accession: phs000920.v1.p1, NHLBI TOPMed: Genes-environments and Admixture in Latino Americans Study). The summary statistics with the whole EWAS results are available at the Zenodo repository: 10.5281/zenodo.5236667. The scripts used for the analyses are available upon request to the corresponding author.

## Abbreviations

BMI: Body Mass Index
COPD: Chronic obstructive pulmonary disease
DMR: Differentially methylated region
DNAm: DNA methylation
EWAS: Epigenome-wide association study
FEV_1_: Forced expiratory volume in 1 s
FVC: Forced vital capacity
GALA II: Study of Genes-Environment and Admixture in Latino Americans
GLI- 12: Global Lung Initiative 2012
MAF: Minor allele frequency
meQTL: Methylation quantitative trait loci
NO_2_: Nitrogen dioxide
O_3_: Ozone
PCs: Principal components
PFT: Pulmonary function test
PM_10_: Particulate matter ≤ 10 µm in diameter
PM_2.5_: Particulate matter ≤ 2.5 µm in diameter
QC: Quality control
RNA: Ribonucleic acid
SHS: Second-hand smoking
SNP: Single nucleotide polymorphism
SO_2_: Sulfur dioxide
US: United States
WGS: Whole-genome sequencing
