# Supplementary figures and images for: Epigenome-wide association study of lung function in Latino children and youth with asthma
Source: Clin Epigenetics. 2022 Jan 15;14:9. doi: 10.1186/s13148-022-01227-5 (PMC8760660; doi:10.1186/s13148-022-01227-5)

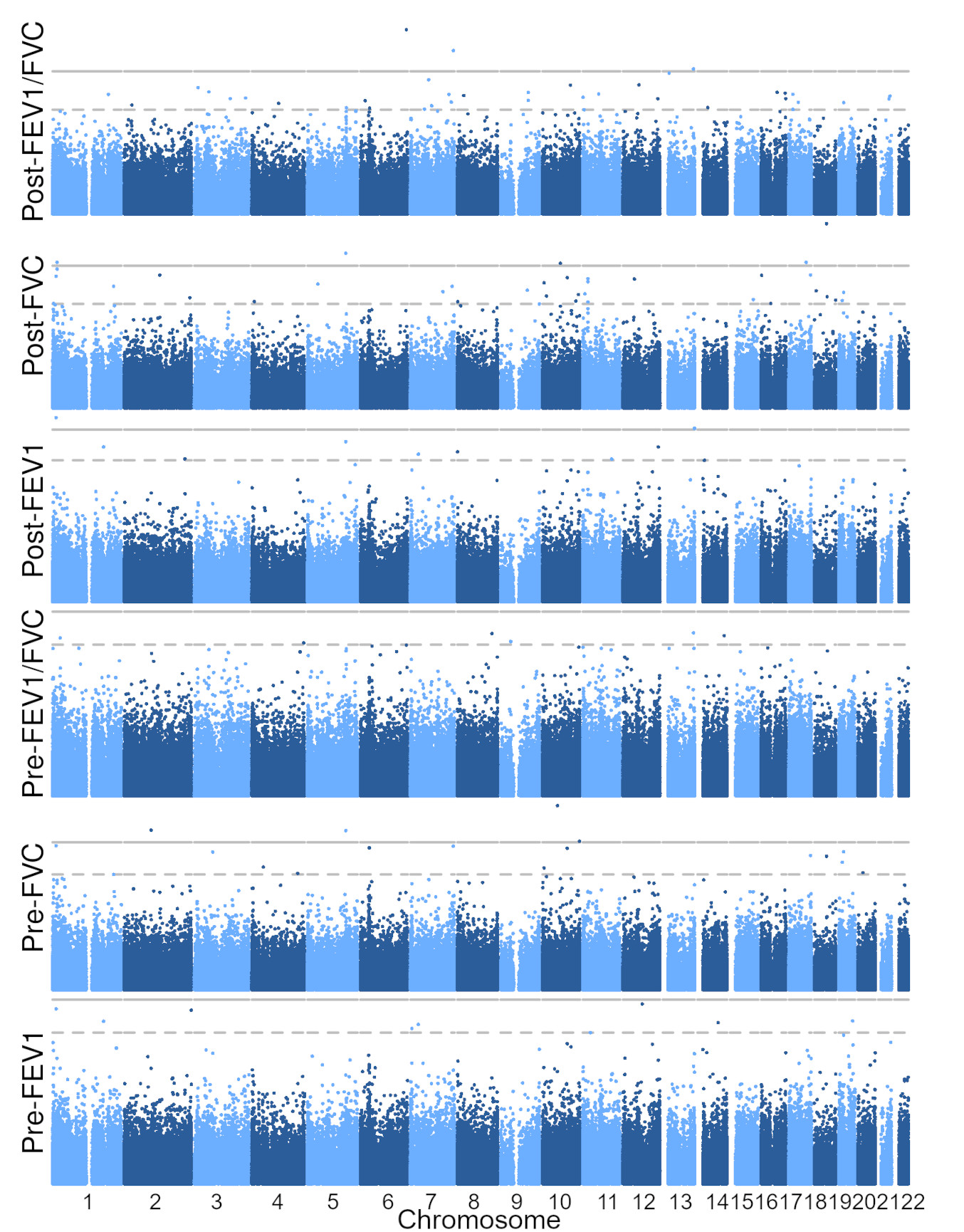

Supplement: Supplementary file 1 — Additional file 1: Fig. S1. Manhattan-plot for the EWAS of lung function in Puerto Ricans. (A) pre-forced expiratory volume in 1 s (FEV1), (B) pre-forced vital capacity (FVC), (C) pre-FEV1/FVC ratio, (D) post-FEV1, (E) post-FVC, and (F) post-FEV1/FVC ratio. The statistical significance of association results (− log10 p value) is represented for each CpG site as a dot (y-axis) along the autosomal chromosomes (x-axis) from chromosome 1 (left) to chromosome 22 (right). The threshold for a false discovery rate less than 1% for each trait of lung function is indicated by the dashed gray line and the genome-wide threshold for significance is represented by the continuous gray line. [file 13148_2022_1227_MOESM1_ESM.jpg]

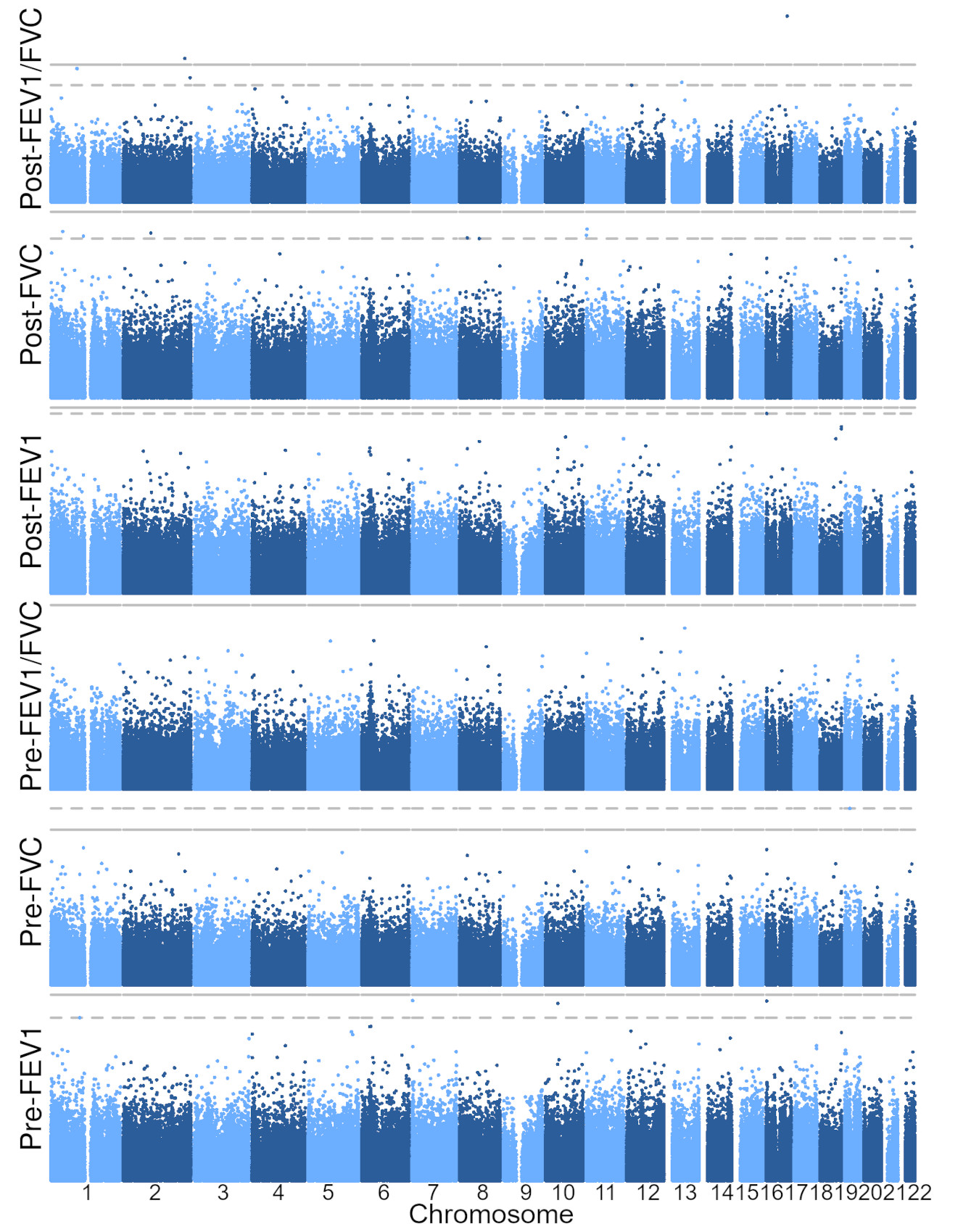

Supplement: Supplementary file 2 — Additional file 2: Fig. S2. Manhattan-plot for the EWAS of lung function in Mexican Americans. (A) pre-forced expiratory volume in 1 s (FEV1), (B) pre-forced vital capacity (FVC), (C) pre-FEV1/FVC ratio, (D) post-FEV1, (E) post-FVC, and (F) post-FEV1/FVC ratio. The statistical significance of association results (− log10 p value) is represented for each CpG site as a dot (y-axis) along the autosomal chromosomes (x-axis) from chromosome 1 (left) to chromosome 22 (right). The threshold for a false discovery rate less than 1% for each trait of lung function is indicated by the dashed gray line and the genome-wide threshold for significance is represented by the continuous gray line. [file 13148_2022_1227_MOESM2_ESM.jpg]

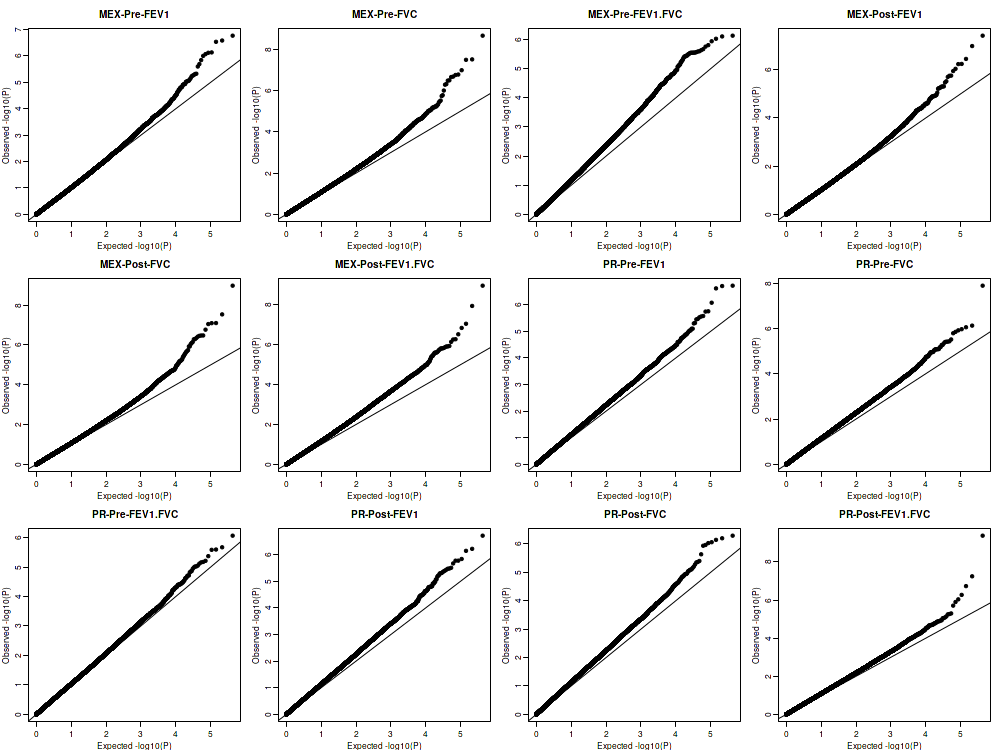

Supplement: Supplementary file 3 — Additional file 3: Fig. S3. Quantile–quantile plot for the EWAS of the association between each specific lung function measurement and DNA methylation in Mexican Americans (MEX) and Puerto Ricans (PR). For the EWAS in Mexican Americans, inflation factors were 1.02, 1.13, 1.24, 1.03, 1.12 and 1.24 for pre-forced expiratory volume in 1 s (FEV1), pre-forced vital capacity (FVC) and their pre-ratio (FEV1/FVC) and post-FEV1, post-FVC and post-FEV1/FVC, respectively. For the EWAS in Puerto Ricans, inflation factors were 1.19, 1.17, 1.09, 1.22, 1.21, 1.20 for pre-FEV1, pre-FVC, pre-FEV1/FVC, post-FEV1, post-FVC and post-FEV1/FVC, respectively. The observed p value (− log10 p value) is shown in the y-axis along the expected p value (− log10 p value) represented in the x-axis. [file 13148_2022_1227_MOESM3_ESM.png]

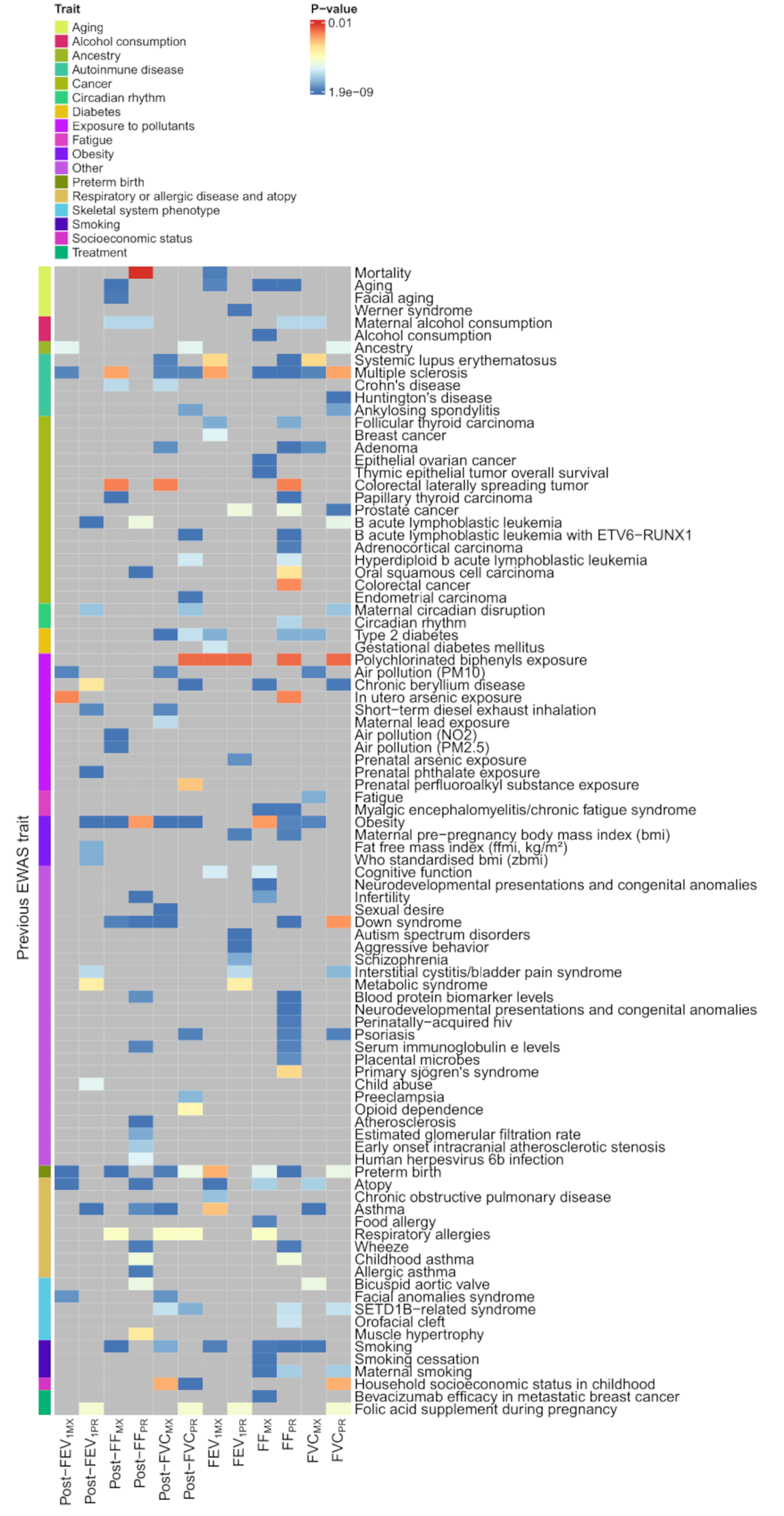

Supplement: Supplementary file 4 — Additional file 4: Fig. S4. Heatmap of the trait enrichment for the top 100 CpGs for each pulmonary function test and ethnicity. Significant results at an FDR < 0.05 for forced expiratory volume in 1 s (Pre-FEV1), forced vital capacity (FVC) and their ratio (FF) pre- and post-administration of albuterol are shown for Puerto Ricans (PR) and Mexican Americans (MX). The enrichment raw p value is colored on a scale from blue (less significant association) to red (more significant association). Non-significant p values are represented in gray. [file 13148_2022_1227_MOESM4_ESM.tif]
