# Supplementary material for: Epigenome-wide association study of lung function in Latino children and youth with asthma
Source: Clin Epigenetics. 2022 Jan 15;14:9. doi: 10.1186/s13148-022-01227-5 (PMC8760660; doi:10.1186/s13148-022-01227-5)
Supplement: Supplementary file 6 — Additional file 6. Supplementary Material. [file 13148_2022_1227_MOESM6_ESM.doc]

**Supplementary material**

**Epigenome-wide association study of lung function in Latino children and youth with asthma**

Esther Herrera-Luis, MSc^1†^, Annie Li^2†^, Angel C. Y. Mak, PhD^2^, Javier Perez-Garcia, PharmD^1^, Jennifer R. Elhawary MSc^2^, Sam S. Oh PhD, MPH^2^, Donglei Hu, PhD^2^, Celeste Eng, BS^2^, Kevin L. Keys, PhD^2,3^, Scott Huntsman, MSc^2^, Kenneth B. Beckman, PhD^4^, Luisa N. Borrell, DDS, PhD^5^, Jose Rodriguez-Santana, MD, FAAP, FCCP^6^, Esteban G. Burchard, MD, MPH^2,7‡^, Maria Pino-Yanes, PhD^‡^*^1,8,9^

^1^Genomics and Health Group, Department of Biochemistry, Microbiology, Cell Biology and Genetics, Universidad de La Laguna, San Cristóbal de La Laguna, Tenerife, Spain

^2^Department of Medicine, University of California San Francisco, San Francisco, California, USA

^3^Berkeley Institute for Data Science, University of California Berkeley, Berkeley, CA, USA

^4^ UMN Genomics Center, Minneapolis, MN, USA

^5^Department of Epidemiology & Biostatistics, Graduate School of Public Health & Health Policy, City University of New York, New York, NY, United States of America

^6^Centro de Neumología Pediátrica, San Juan, Puerto Rico.

^7^Department of Bioengineering and Therapeutic Sciences, University of California San Francisco, San Francisco, California, USA

^8^CIBER de Enfermedades Respiratorias, Instituto de Salud Carlos III, Madrid, Spain

^9^Instituto de Tecnologías Biomédicas (ITB), Universidad de La Laguna, San Cristóbal de La Laguna, Tenerife, Spain

^†^ Equal contribution as first authors

^‡^Equal contribution as senior authors

***Corresponding author:**

Dr. Maria Pino-Yanes

Genomics and Health Group. Department of Biochemistry, Microbiology, Cell Biology and Genetics. Universidad de La Laguna

Apartado 456, 38200, San Cristóbal de La Laguna, Santa Cruz de Tenerife, Spain

Phone: (+ 34) 922 316502 6343

Fax: (+ 34) 922 318 490

e-mail: [mdelpino@ull.edu.es](mailto:mdelpino@ull.edu.es)

Supplementary Methods

**Study participants**

GALA II is an ongoing case-control study of pediatric asthma in Hispanics/Latinos that were recruited between 2006-2014 in five areas from the US (Chicago, Bronx, Houston, San Francisco Bay Area) and Puerto Rico (San Juan)^1,2^. Within that period of time, some of the recruited individuals were summoned for a follow-up to collect additional biological samples and the corresponding updated questionnaire data. Briefly, individuals were included if they were a between 8-21 years old, self-identified as Hispanic or Latino, and had four Latino grandparents. Asthma was defined by physician diagnosis, use of controller or rescue medication, and report of two or more symptoms of coughing, wheezing, or shortness of breath. Exclusion criteria were any of the following: (1) 10 or more pack-years of smoking; (2) any smoking in the year preceding recruitment; (3) history of lung diseases other than asthma (cases) or chronic illness (cases and controls); or (4) pregnancy in the third trimester.

**Pulmonary function tests**

Pre- and post- bronchodilation spirometric data for forced expiratory volumen in 1 second (FEV_1)_, forced vital capacity (FVC) and their ratio was recorded with a KoKo® PFT Spirometer (nSpire Health Inc., Louisville, CO) according to American Thoracic Society recommendations (1995)^3^. Subjects with asthma were instructed to withhold their bronchodilator medications for at least 8 hours prior to pulmonary function testing. Post-bronchodilator PFT values were measured 15 minutes after providing the participants a dose of albuterol, consisting of 6 (if aged<16 years) to 8 (if aged ≥16 years) puffs of albuterol. Individuals with implausible data (FEV_1_/FVC>1) or that deviated 1.5*Interquartile range, for each ethnic group, were excluded from further analysis. Percent predicted were normalized against predicted values from the Global Lung Initiative 2012 (GLI12) reference equations^4^.

**Air pollution data acquisition**

Details on the assessment of air pollution exposure has been described elsewhere^2^. Briefly, average environmental exposure to ozone, nitrogen dioxide, sulfur dioxide, particulate matter not greater than 2.5 µm in diameter, and particulate matter not greater than 10 µm in diameter were estimated from the data from the four closest monitoring stations to the participants residence. Average lifetime exposures were estimated using available yearly average estimates over the lifetime of the participant until the day of spirometry testing.

**Methylation profiling and quality control**

DNA methylation measurements were obtained from whole blood using the InfiniumEPIC BeadChip or the Infinium HumanMethylation450 BeadChip (Illumina, San Diego, CA) array. Sampling of individuals that were previously profiled with the Infinium HumanMethylation450 BeadChip array included children with asthma and *in utero* smoking exposure, and some who were exposed to secondhand smoking (SHS), as well as unexposed control subjects with neither *in utero* nor current SHS. Subjects with *in utero* smoking exposure were matched with unexposed subjects on age and gender. For those individuals to be profiled with the EPIC array, priority was given to subjects with smoking exposure data (current smokers, exposed or unexposed to secondhand smoking and *in utero* smoking), inhaled corticosteroid use, and existing omics data, including whole genome and RNA sequencing data. Exposed and unexposed subjects were matched by asthma status, age, and sex.

Briefly, 1ug of DNA was bisulfite-converted using the Zymo EZ DNA Methylation Kit (Zymo research, Irvine, CA) and manufacture protocols were followed for both arrays. Low quality probes (beads<3 or a detection p-value>1x10^-6^ for ≥5% of the samples) and samples with low quality data points for ≥5% of the CpG sites were removed along with samples with a total bisulfite intensity less than 3 standard deviations of the sample bisulfite control. We further filtered outliers of the total bisulfite intensity or beta value distribution. We then performed "oob" background correction^5^, "RELIC" byas correction^6^, inter-array ("quantile1") normalization, and Regression on Correlated Probes probe-type (RCP) bias adjustment^7^. After removal of outliers of intensity values and beta-value distribution, samples with more than 10% of missing probes, as well probes with missing values in more than 5% of the samples, missing values were imputed. We further filtered samples with mismatched sex and probes on sex chromosomes. Moreover, individuals with or mixed genotype distributions on the control single nucleotide polymorphism (SNP) probes were identified with ewastools R package (v1.7)^8^ and excluded. For downstream analyses, we included those CpG probes that were not identified as cross-reactive probes^9,10,11^, CpG sites containing a single nucleotide polymorphism (SNP) with minor allele frequency > 1% at the CpG site or at a single base extension and multimodal probes identified with Enmix (1.22.0)^5^ R package.

**Statistical analysis**

Genetic principal components (PCs) and genetic relatedness were computed using biallelic SNPs with a PASS flag from TOPMed freeze 8 DP10 data by means of the the PC-Relate function from the GENESIS R package^12,13^ using a workflow available from the Summer Institute in Statistical Genetics Module 17 course website^14^. For the methylation quantitative trait loci analysis, a set of independent variants was identified by linkage disequilibrium clumping (prunning of SNPs with pairwise *r^2^* < 0.25 within 250 kilobases) using PLINK 1.9^15^. Differentially methylated regions were assessed using the uncorrected p-values from the EWAS with comb-p^16^. The seed P value was set to 0.05. To define a significant region, a minimum number of 2 CpGs, a maximum 1 distance of 1000 bp and Sidak-corrected p-value of 0.05 were required.

**References**

1. Nishimura KK, Galanter JM, Roth LA, Oh SS, Thakur N, Nguyen EA, et al. Early-life air pollution and asthma risk in minority children. The GALA II and SAGE II studies. Am J Respir Crit Care Med. 2013;188(3):309-18.
2. Thakur N, Oh SS, Nguyen EA, Martin M, Roth LA, Galanter J, et al. Socioeconomic status and childhood asthma in urban minority youths. The GALA II and SAGE II studies. Am J Respir Crit Care Med. 2013;188(10):1202-9.
3. Standardization of Spirometry, 1994 Update. American Thoracic Society. Am J Respir Crit Care Med. 1995;152(3):1107-36.
4. Lytras, T. rspiro: Implementation of spirometry equations. R package version 0.2. 2020.
5. Xu Z, Niu L, Li L, Taylor JA. ENmix: A novel background correction method for Illumina HumanMethylation450 BeadChip. Nucleic Acids Res. 2016;44(3):e20.
6. Xu Z, Langie SAS, De Boever P, Taylor JA, Niu L. RELIC: A novel dye-bias correction method for Illumina Methylation BeadChip. BMC Genomics. 2017;18:4.
7. Niu L, Xu Z, Taylor JA. RCP: a novel probe design bias correction method for Illumina Methylation BeadChip. Bioinformatics. 2016;32(17):2659-63.
8. Heiss JA, Just AC. Identifying mislabeled and contaminated DNA methylation microarray data: An extended quality control toolset with examples from GEO. Clin Epigenetics 2018;10:73.
9. Pidsley R, et al. Critical evaluation of the Illumina MethylationEPIC BeadChip microarray for whole-genome DNA methylation profiling. Genome Biol. 2016;17(1):208.
10. Price ME, Cotton AM, Lam LL, Farré P, Emberly E, Brown CJ, et al. Additional annotation enhances potential for biologically-relevant analysis of the Illumina Infinium HumanMethylation450 BeadChip array. Epigenetics Chromatin. 2013;6(1):4.
11. Chen YA, Lemire M, Choufani S, Butcher DT, Grafodatskaya D, Zanke BW, et al. Discovery of cross-reactive probes and polymorphic CpGs in the Illumina Infinium HumanMethylation450 microarray. Epigenetics. 2013;8(2):203-9.
12. Conomos MP, Miller MB, Thornton TA. Robust inference of population structure for ancestry prediction and correction of stratification in the presence of relatedness. Genet Epidemiol. 2015;39(4):276-93.
13. Conomos MP, Reiner AP, Weir BS, Thornton TA. Model-free Estimation of Recent Genetic Relatedness. Am J Hum Genet. 2016;98(1):127-48.
14. Summer Institute in Statistical Genetics, PC-Relate. 2019. <https://uw-gac.github.io/SISG_2019/pc-relate.html>. Accessed 21 June 2020.
15. Chang CC, Chow CC, Tellier LCAM, Vattikuti S, Purcell SM, Lee JJ. Second-generation PLINK: Rising to the challenge of larger and richer datasets. Gigascience 2015;4:7.
16. Pedersen BS, Schwartz DA, Yang IV, Kechris KJ. Comb-p: software for combining, analyzing, grouping and correcting spatially correlated P-values. Bioinformatics. 2012;28(22):2986-8.

Supplementary Figure Legends

**Figure S1. Manhattan-plot for the EWAS of lung function in Puerto Ricans.** A) pre-forced expiratory volume in 1 second (FEV_1_), B) pre-forced vital capacity (FVC), C) pre- FEV_1_/FVC ratio, D) post-FEV_1_, E) post-FVC, and F) post- FEV_1_/FVC ratio. The statistical significance of association results (-log_10_ *p*-value) is represented for each CpG site as a dot (*y*-axis) along the autosomal chromosomes (*x*-axis) from chromosome 1 (left) to chromosome 22 (right). The threshold for a false discovery rate less than 1% for each trait of lung function is indicated by the dashed grey line and the genome-wide threshold for significance is represented by the continuous grey line.

Figure S2. Manhattan-plot for the EWAS of lung function in Mexican Americans. A) pre-forced expiratory volume in 1 second (FEV_1_), B) pre-forced vital capacity (FVC), C) pre- FEV_1_/FVC ratio, D) post-FEV_1_, E) post-FVC, and F) post- FEV_1_/FVC ratio. The statistical significance of association results (-log_10_ *p*-value) is represented for each CpG site as a dot (*y*-axis) along the autosomal chromosomes (*x*-axis) from chromosome 1 (left) to chromosome 22 (right). The threshold for a false discovery rate less than 1% for each trait of lung function is indicated by the dashed grey line and the genome-wide threshold for significance is represented by the continuous grey line.

**Figure S3. Quantile–quantile plot for the EWAS of the association between each specific lung function measurement and DNA methylation in Mexican American (MEX) and Puerto Ricans (PR).** For the EWAS in Mexican Americans, inflation factors were 1.02, 1.13, 1.24, 1.03, 1.12 and 1.24 for pre-forced expiratory volume in 1 second (FEV_1_), pre-forced vital capacity (FVC) and their pre-ratio (FEV1/FVC) and post-FEV_1_, post-FVC and post-FEV_1_/FVC, respectively. For the EWAS in Puerto Ricans, inflation factors were 1.19, 1.17, 1.09, 1.22, 1.21, 1.20 for pre-FEV1, pre-FVC, pre-FEV1/FVC, post-FEV_1_, post-FVC and post-FEV_1_/FVC, respectively. The observed p-value (-log_10_ *p*-value) is shown in the *y*-axis along the expected *p*-value (-log_10_ *p*-value) represented in the *x*-axis.

**Figure S4. Heatmap of the trait enrichment for the top 100 CpGs for each pulmonary function test and ethnicity.** Significant results at an FDR<0.05 for forced expiratory volume in 1 second (Pre-FEV_1_), forced vital capacity (FVC) and their ratio (FF) pre- and post- administration of albuterol are shown for Puerto Ricans (PR) and Mexican Americans (MX). The enrichment raw p-value is colored on a scale from blue (less significant association) to red (more significant association). Non-significant *p*-values are represented in grey.
